# Editorial: Molecular and cellular players of axonal regeneration in injured CNS

**DOI:** 10.3389/fnins.2023.1315632

**Published:** 2023-11-01

**Authors:** Ana Raquel Santiago, Inês Dinis Aires, Marta Agudo-Barriuso, Raquel Boia

**Affiliations:** ^1^University of Coimbra, Coimbra Institute for Clinical and Biomedical Research (iCBR), Faculty of Medicine, Coimbra, Portugal; ^2^University of Coimbra, Center for Innovative Biomedicine and Biotechnology (CIBB), Coimbra, Portugal; ^3^Clinical Academic Center of Coimbra (CACC), Coimbra, Portugal; ^4^Association for Innovation and Biomedical Research on Light and Image (AIBILI), Coimbra, Portugal; ^5^University of Coimbra, Institute of Immunology, Faculty of Medicine, Coimbra, Portugal; ^6^Grupo de Oftalmología Experimental, Departamento de Oftalmología, Optometría, Otorrinolaringología y Anatomía Patológica, Facultad de Medicina, Instituto Murciano de Investigación Biosanitaria (IMIB), Universidad de Murcia, Murcia, Spain

**Keywords:** injured central nervous system, axonal regrowth, axonal regeneration, optic nerve regeneration, functional recovery

Central nervous system (CNS) has a limited regenerative capability, which makes it difficult to repair after injury. Damage of axons from CNS, as in spinal cord injury, traumatic brain injury, stroke and optic neuropathies, leads to permanent functional deficits, due to axonal disconnection. Multiple factors, including the decline of intrinsic growth capacity, the inhibitory extrinsic environment, and neuronal vulnerability after lesion, contribute to regenerative failure in the adult injured CNS. However, the concept that axons from CNS neurons are not able to regenerate after an injury was challenged after the demonstration that those axons can grow into peripheral nerve grafts (Richardson et al., 1980; David and Aguayo, 1981). Thus, a deeper understanding on the intrinsic and extrinsic molecular pathways that limit or promote axon regeneration may pinpoint new therapeutic targets and will contribute to the development of better therapeutic approaches for CNS injury. Indeed, several studies have arisen attempting to find means to overcome the barriers to axon regrowth. A logical repair strategy would first promote regeneration of severed axons by providing a permissive environment in order to restore structure and function of the damaged axons.

Despite the increased knowledge over the years, there is currently no approved treatments aiming to promote CNS axon growth and regeneration. This Research Topic aimed at widening the knowledge in the structural and functional restoration of the severed axonal processes in the CNS.

Upon a CNS injury, the formation of a glial scar is the key event that creates a non-permissive environment for axonal regeneration. Thus, Costa et al. deeply reviewed the implications of glial scar formation for axonal regeneration and discussed the key factors impeding axonal growth after injury. The authors first described the mechanism of axonal regeneration in the peripheral nervous system (PNS), a context with higher potential for regeneration, in order to gain insights on the potential mechanisms within CNS. Moreover, in this article it was also included a comprehensive review of promising new therapeutic targets in eliciting CNS axonal regeneration.

As the astrocytes are one cellular component of the scar tissue, being activated after axonal injury and proliferating to form an astrocytic scar border commonly associated with inhibition of axon regeneration, Hemati-Gourabi et al. dissected the dual role of glial scar in the pathological process of spinal cord injury. In this context, the authors described the controversial role of the glial scar over time and explored the underlying mechanisms of its diverse and dynamic nature, with a focus on the disparity, variation, and interactions of scar-forming astrocytes. In this article it was reviewed the neuroprotective effect driven by astrocytic scar in the early phase of acute focal injury and the positive role of astrocytes in axon regeneration and axon sprouting in the mature mammalian CNS. The potential mechanisms underlying axon growth-supportive effects of astrocytes were reviewed in detail, and include the production of neurotrophic factors, remodeling of the extracellular matrix, clearance of myelin debris, and provision of bioenergetic support for axon growth.

Fu et al. explored in their comprehensive review the impressive progress in the biological, engineering and rehabilitation strategies for repairing the injured spinal cord. The authors focused on the use of Schwann cells transplantation to promote spinal cord injury repair, that has shown encouraging results in animal models by enhancement of axon regeneration, remyelination of newborn or sparing axons, regulation of the inflammatory response, and maintenance of the survival of damaged tissue, that leads to functional recovery. Some of these Schwann cells transplantation therapies have been subject to phase I clinical trials, which have confirmed their safety and feasibility for the treatment of spinal cord injury, however little functional improvement was observed. Despite the immense knowledge gained through the years, there is currently no approved treatment for clinical use in spinal cord injury patients. The authors also discuss the challenges in translating Schwann cells transplantation into clinical practice, and argued the complex pathophysiologic mechanisms of spinal cord injury and the marked differences between the animal and human spinal cord as the main barriers for this translation.

In this way, Lear and Moore focused their review paper on the advantages and disadvantages of the use of human neuronal model systems to study axon regeneration with the goal of bridging basic science studies into clinical trials. Tremendous progress has been made in identifying intrinsic and extrinsic regulators of CNS axon regeneration in rodents, however it is not known yet if these are conserved in human CNS neurons. This makes it extremely important to understand if CNS axon growth and regeneration ability also occurs in a context of human model systems. The authors described that the advent of human pluripotent stem cell (hPSC) technology, which includes both human embryonic stem cells (hESCs) and induced pluripotent stem cells (iPSCs), and direct reprogramming technologies, allow to study axon growth and regeneration in human disease and therapeutic models. Thus, the use of human systems to study CNS axon growth and regeneration may accelerate and increase the success of the transition of preclinical studies to clinical trials.

## Author contributions

AS: Writing – review & editing. IA: Writing – review & editing. MA-B: Writing – review & editing. RB: Writing – original draft, Writing – review & editing.
